# The Next Frontier in Health Geography: Context and Implications for Interventions

**DOI:** 10.3390/ijerph16081457

**Published:** 2019-04-24

**Authors:** Andrew Curtis, Jacqueline Curtis

**Affiliations:** Department o Population and Quantitative Health Sciences, School of Medicine, Case Western Reserve University, Cleveland, OH 44106, USA; jcurti21@kent.edu

During an opening panel of the 2018 International Health Congress in Oxford, a question was raised as to how protection could be offered to young researchers in particular who explore new ideas. It was argued that often grant review panels, journal editors, and reviewers crushed new ideas or approaches simply because they did not conform to the norms of existing research. These gatekeepers are more secure in approving studies that make minute advances on existing lines of inquiry, rather than expose a journal or agency to the potential critiques that can come with truly novel perspectives. We have certainly experienced this with some of our past papers, especially those in a series of new methods or topical approaches. Indeed, we have had conversations with editors who have told us that key papers, for example, the first proposing a new method that has gone on to revolutionize an area, were initially rejected because reviewers and editors were too conservative. Certainly, there will always be ideas that are not suitable for publication, such as those with faulty approaches, however, we believe we need to provide more opportunities for exploration. In the United States, where funding amounts or numbers of publications dictate promotion or even job security, venturing down this path can be a terrifying prospect for university faculty. However, it is our position that without pursuing this type of inquiry, the great step changes in understanding and improving health will not occur as quickly as they are needed. 

One of the main reasons for our development of this Special Issue was to give a forum for more exploratory work. The second reason was to focus on two important areas emerging in the spatial sciences: fine spatial scale research and the importance of geographic context. Investigations of fine and dynamic geographic scales are essential to understanding the interaction of processes that are not captured with existing static official data, and then to developing place-based solutions. This is what we call working at “the scale of intervention”. For example, if we assign census data to our cases, or map events by tract, zip code etc., what does that really tell us? For the teenage sexually transmitted infection cluster we need to know activity space rather than home residence. For access to food, we need to identify the barriers, physical, cultural or perceived that shape this issue. Then, we need output that can be used by those on the front line. Regarding the geographic context of these places, processes, and outcomes, we want to provide an opportunity for scholars to expand the groundbreaking work of Mei Po Kwan, which has paved the way for the studies presented in this Special Issue through her identification of the uncertain geographic context problem (UGCoP) [1]. This body of research argues for re-evaluation to determine if we have the correct data, and if not, how do we acquire it? In addition, there is the added value of expert interpretation, not only from an academic perspective but also from those who know their own environments. 

It is with these intentions that we offer the seven papers that comprise this Special Issue. They represent various environments and settlement types, and range in focus from the physical environment to social determinants of health. They highlight numerous challenges in infectious and chronic disease control and prevention, and offer practical geospatial solutions through new forms of data collection, analysis, as well as new publicly available software applications. Three of these papers are based in U.S., while the other four focus on global health. In the U.S., Bell et al. contribute new insights into obesity and obesogenic environments through a focus on the role of racial inequalities in socioeconomic status. Shook et al. offer proof of concept approach on risk perception and behaviors captured in the midst of potential ebola exposure. In a novel technology-focused piece, Ajayakumar et al. present new approaches for collecting and analyzing geonarratives through a case study of post-disaster recovery after a catastrophic tornado. In the area of global health, Krystosik et al. highlight new spatial documentation of the role of neighborhood violence in arboviral surveillance and control. Two papers by Curtis et al. demonstrate using spatial video to collect new forms of data in challenging environments, especially in informal settlements. These studies highlight the complex human-environmental interactions that comprise the dynamic and micro-geographic processes of disease risk. Finally, building on these global health studies, work by Blackburn et al. concludes this Special Issue by modelling environmental transmission of diseases, with a focus on local infectious zones (LIZs) and their dynamic temporal dimensions in disease transmission. In different ways, these seven papers delve into that risky, but potentially rewarding, exploratory territory we described at the beginning of this editorial. As a result of their willingness to explore new frontiers of data collection, analysis, and even new technology and conceptual/theoretical frameworks, we believe that each has made a valuable contribution that advances knowledge for improved health outcomes.

In summary, the articles in this Special Issue contribute to a growing body of evidence that points to the complexity of geographic contexts in shaping health outcomes, and the implications for interventions. Despite the awareness of this complexity, it is challenging for spatial investigations to fully account for its presence. For example, there is a dearth of guidance on the identification and representation of the dynamic spatial and temporal scales that often interact to yield a particular outcome. In addition to scale, questions are emerging about the suitability of “official” data, the potential role for local knowledge or other “unofficial” data, and how these potentially disparate sources can be meaningfully integrated in a geographic information system. To address such challenges, a new frontier of health geography is emerging. This frontier is focusing on new forms of geospatial technologies, novel methods and analytical approaches including customized software development, and a means to capture physical and social context. This Special Issue has been developed to showcase new methods and applications that can be applied to a wide variety of health problems, in any health setting.
